# Building breastfeeding knowledgeable health systems: Focus groups with physician leaders

**DOI:** 10.1371/journal.pone.0350146

**Published:** 2026-05-28

**Authors:** Miena Meek Hall, Julie A. Patterson, Alexandra L. MacMillan Uribe, Liliana Simon, Anne R. Eglash, Katherine R. Standish

**Affiliations:** 1 Department of Family Medicine and Community Health, School of Medicine and Public Health, University of Wisconsin, Madison, Wisconsin, United States of America; 2 Mothers’ Milk Bank of the Western Great Lakes, Elk Grove Village, Illinois, United States of America; 3 School of Health Studies, College of Health and Human Sciences, Northern Illinois University, DeKalb, Illinois, United States of America; 4 Institute for Advancing Health Through Agriculture, College of Agriculture and Life Sciences, Texas A&M University, College Station, Texas, United States of America; 5 Department of Pediatrics, School of Medicine, University of Maryland, Baltimore, Maryland, United States of America; 6 Department of Family Medicine, Chobanian & Avedisian School of Medicine, Boston University‌‌, Boston, Massachusetts, United States of America; Medical Research Council, SOUTH AFRICA

## Abstract

Despite the growth of breastfeeding and lactation medicine as a specialty, the care of breastfeeding families is compromised because few standards and recommendations exist for its practice and integration into health systems. We conducted a qualitative study involving three focus groups (N = 13) with breastfeeding and lactation medicine physician leaders who currently work within large health systems across the United States. Our study aimed to gather the perspectives of physician leaders regarding breastfeeding care in health systems, to summarize current practices and recommendations for optimal care, and to explore barriers and facilitators to the implementation of these recommendations. A deductive content analysis approach guided by the Exploration, Preparation, Implementation, and Sustainment Framework was used to analyze the transcripts. Resulting themes revealed the important role that health systems play in modeling breastfeeding supportive practices and the strong influences of leadership and staff personal breastfeeding experiences on health system policies. Recommendations included the creation of breastfeeding and lactation medicine divisions, adequate staff education, staffing and coordination of lactation care across the system and community, support for lactating employees, and public awareness of resources and programs. Barriers to implementation included siloing of lactation services by department, lack of breastfeeding-supportive workplaces, deficient clinical billing for lactation services, and low prioritization by training programs. Facilitators included multidisciplinary collaborations, employee supportive lactation policies, appropriate dyadic lactation billing, and electronic health record workflows. Our focus groups revealed many barriers to the delivery of optimal breastfeeding care within health systems, but strategies were identified for systemic changes. Next steps include identification of breastfeeding and lactation medicine divisions and health systems already implementing the best practices described here. Further research should engage additional stakeholders to better understand administrative and financial points of view regarding barriers and facilitators of breastfeeding support.

## Introduction

Breastfeeding is normative infant feeding and associated with lower risk of infant morbidity and mortality as compared to formula feeding [1]. Although birth parents in the United States have a high intention to breastfeed, with over 84% initiating breastfeeding, exclusive breastfeeding rates drop off rapidly, with only 25% of infants exclusively breastfeeding at 6 months of age [2,3]. Shorter breastfeeding duration or lack of exclusivity increases lifelong health risks for mother-infant dyads and may contribute to postpartum depression, anxiety, and feelings of guilt or failure for the lactating parent [4]. Although all infants benefit from breastfeeding, it is critical for infants with medical vulnerabilities, such as prematurity, low birth weight, congenital heart defects, prenatal opioid exposure, metabolic diseases, and genetic diseases, to be fed human milk [5,6]. These children are often cared for in health systems that may not adequately support breastfeeding or do not use donor milk when parental milk is unavailable [5–7]. Suboptimal breastfeeding practices across all infant populations result in significant social and economic costs, estimated at US$100 billion annually in the US [8].

Multiple systemic barriers, beyond personal control, prevent families from meeting their breastfeeding intentions. Leading medical organizations, including the US Preventive Services Task Force, American Academy of Family Physicians, American College of Obstetricians and Gynecologists, and American Academy of Pediatrics, strongly recommend routine breastfeeding counseling and support [1,9–11]. Breastfeeding care, however, is poorly integrated into healthcare, with many health professionals lacking adequate education and training, leading to insufficient lactation support ultimately contributing to a decline in breastfeeding rates over time. [12,13]. While some outpatient pediatric [14,15] and obstetrics [16,17] clinics offer breastfeeding support, it is not widely implemented.

At the same time, physician employment within the US is increasingly concentrated within large multispecialty health systems. In 2023, 77.6% of physicians were employed by hospitals or health systems, up from 25.8% in 2012 [18]. Breastfeeding families interact with multiple departments in these systems, yet support typically ends after the short, one-to-three day inpatient stay following delivery [19]. Many hospitals have adopted initiatives such as the Baby Friendly Hospital Initiative (BFHI) [20,21] to improve breastfeeding rates during the immediate postpartum period [22]. However, systematic integration of follow-up breastfeeding support into outpatient care remains limited, access to a higher level of care for complex cases is lacking in many communities [23], and evidence-based strategies are not widely shared. Policies and infrastructure needed to support breastfeeding for two years and beyond [24] have yet been to be fully developed [25,26]. Lack of physician and provider education and medical system breastfeeding support has been found to be a barrier in the continuation of care in breastfeeding support [27].

In recognition for the need to better support lactating families within health systems, Breastfeeding and Lactation Medicine (BFLM) has emerged as a subspecialty. New fellowship and organizational training programs and board certification [28–31] have helped define and formalize the field. BFLM clinics, programs and academic divisions have launched efforts to improve care, but standard and broad implementation is lacking [32–34]. To bridge these critical gaps, we conducted a qualitative study with BFLM physician leaders exploring their perspectives on the implementation of optimal breastfeeding care in health systems, including barriers, facilitators and recommendations.

## Materials and methods

### Study design‌‌

Qualitative focus groups were conducted with BFLM physicians working within large health systems in the US to explore their perspectives on 1) the role and rationale of health systems engaging in breastfeeding support and care, 2) current health systems’ practices, 3) recommendations for developing breastfeeding knowledgeable health systems (BKHS), and 4) barriers and facilitators to implementing such changes. For this study, large health systems were defined as multispecialty practices within a hospital system or academic medical center. The Consolidated Criteria for Reporting Qualitative Studies guided study design and reporting [35].

### Framework

This study was guided by the Exploration, Preparation, Implementation, and Sustainment (EPIS) framework, which influenced the development of the focus group questions and qualitative analysis. The framework provides a comprehensive approach to understanding and guiding the implementation of evidence-based practices through four distinct phases: Exploration, Preparation, Implementation, and Sustainment [36]. This study focused on the Exploration and Preparation phases to identify needs, define core tenets of the innovation (i.e., characteristics of BKHS), and identify barriers and facilitators to implementation. Globally, EPIS has been applied across a wide range of public, social, and allied health service systems to investigate the implementation of a specific evidence-based practice [37]. The EPIS framework includes several key constructs that influence the implementation process: 1) inner context that encompasses an organization’s characteristics (e.g., leadership organizational structure, individual characteristics); 2) outer context that is external to the organization and includes recipients’ characteristics (e.g., pregnant and lactating individuals and the breastfeeding infant); 3) bridging factors that identify relationships between the inner and outer domain; and 4) innovation factors that characterize the innovation itself. Definitions, EPIS domains, and constructs are in S1 Table.

### Focus group guide

The focus group guide was developed by study team members who were 1) similar to study participants in having extensive experience with BFLM in large health systems, and 2) were able to ascertain the terminology and language context used in the focus group were consistent with the field. Iterative modifications refined the guide to address emerging topics. Open-ended questions and probing techniques encouraged in-depth discussions. Focus group questions are included in S2 Table.

### Participants and recruitment

We used a purposeful critical case sampling approach, identifying key experts who can reveal critical patterns, issues, and themes within a niche phenomenon [38]. We identified BFLM physicians as critical cases (experts) to provide the most information and understanding of how to develop and implement BKHS because of their deep knowledge of patients’ lactation-related needs and the diversity of their experiences implementing best practices in breastfeeding within health systems. Inclusion criteria were: 1) adults over 18 years old, 2) physicians who practice BFLM within a large, multi-specialty health system, and 3) physicians who function as leaders in BFLM at their institutions and have experience implementing clinical and/or policy changes in their health system. To identify individuals who met inclusion criteria, study team members KS and AE identified eligible BFLM programs based on their professional networks and relationships in the BFLM field, and among them selected individuals for whom BFLM comprised a substantial portion of their professional responsibilities (e.g., a neonatologist implementing human milk-focused programs).

Participants were recruited through personal email invitations and interested individuals were asked to reply to be enrolled and scheduled for one of three focus groups from 01/09/2022–31/10/2022. The resulting focus group participants had a professional relationship with KS and AE due to their shared disciplines as BFLM physicians, a subspecialty with a small number of physicians in the US. Our objective was to gather strategic and implementation-relevant insights and expert perspectives from physician leaders. The Institutional Review Boards (IRB) of Northern Illinois University and Boston University approved this study as Exemption Category 2, which permits the use of oral consent when written documentation is not required and may increase risk. Because the BFLM community is small and professionally identifiable, obtaining written consent would have created additional identifiers; therefore, oral consent was deemed the most protective approach.

### Data collection

Before starting the recording, verbal consent to participate in the online focus group and permission to record the session were obtained. Verbal consent was deemed sufficient due to the minimal risk to participants posed by online focus groups regarding professional activities and due to the added logistical complexity of obtaining written consent in a virtual setting. Participants in the online focus groups could choose whether and what information they would like to share and were permitted to leave the group at any time. Their verbal consent was documented in a protected record, noting the date and time, confirming that consent was obtained and was witnessed by the three research team members who led and moderated the focus groups (JP, KS, and AE), per IRB approval.

Focus groups were conducted using video conferencing software and audio- and video-recorded using the software features (Zoom, version 5.12.0 [Zoom Video Communications, Inc., 2022, San Jose, CA, USA]). Each focus group lasted 60–90 minutes. Two research team members led and moderated the focus groups (KS and AE); other study team members (KS, AE, and JP) actively participated in the discussion. Additionally, one team member (JP) took notes during each discussion. Following the focus groups, participants were asked to fill out a brief, anonymous, demographic survey. Subjects did not receive any compensation or incentives for their participation.

### Data analysis

Audio-recorded focus group discussions were transcribed verbatim and checked for errors by JP and KS. Participant confidentiality was safeguarded by the de-identification of all transcripts, including the removal of names, institutions, geographic locations, and other potentially identifying details. De-identified quotations were then categorized by focus group number (1–3) and uploaded to NVIVO version 1.7.1 (QSR International Pty Ltd., Burlington, MA, USA) to assist with data organization and analysis. Given the limited number of BFLM physicians in the US, data saturation, defined as consistent responses or exhaustion of possible responses to the primary research question, was not determined [39].

Transcripts were analyzed using a directed content analysis approach [40] in which EPIS was used to develop an initial deductive codebook. Initially, all study team members read the transcripts. Two team members, KS and JP, coded all transcripts using deductive codebook. As codes were applied using line-by-line coding, the research team met regularly to build consensus around the codebook, during which inductive codes and code sub-categories were created and added to the codebook. Once all transcripts were coded, any deductive codes (i.e., from EPIS) that proved minimally relevant were dropped or revised prior to finalizing codebook. The final codebook included 1) deductive codes informed by EPIS; 2) inductive codes created for relevant data that did not align with EPIS; and 3) code sub-categories of each of the former to further reflect emerging ideas. Coded segments pertaining to each code were reviewed by KS, JP, and MH who grouped together relevant segments to identify emerging themes. Emerging themes were then discussed with the full team until consensus was reached.

The emerging themes were organized by EPIS domains to build final themes, which then served as the foundation for the development of the eight recommendations by the full team. These recommendations were then refined and finalized through team discussions. Member checking with focus group participants was performed via online survey to validate and confirm agreement with finalized recommendations. Memos and notes were created during every meeting to document the process. S3 Table includes codes used in analysis across EPIS Domains.

### Trustworthiness

Trustworthiness addressing the findings’ credibility, transferability, and confirmability was obtained through multiple strategies, including the use of multiple coders and team-based data analysis. To enhance credibility, two research team members independently coded all transcripts (KS and JP), and all members participated in consensus-building meetings in which excerpts within each code were discussed in depth, contributing to theme construction. Expert triangulation was conducted with other BFLM physicians and midwives (n = 5) to review preliminary findings including the 8 recommendations. They agreed with the findings and did not identify any missing themes. Transferability was addressed through purposive sampling, in which critical cases were recruited as participants, and a thick description of study results, allowing readers to identify transferability to other contexts.

### Description of research team

Six members formed the research team: four practicing physicians and two PhD researchers. Of the physicians, two are BFLM clinicians with research experience (KS, AE), one is a pediatric intensivist with health systems breastfeeding implementation expertise (LS), and the fourth is a BFLM physician gaining research training at the post-doctoral fellowship level (MH). The doctoral researchers, JP and LMU, have training and expertise in behavioral research, qualitative methods, and infant feeding. KS, AE, and JP designed the original study. MH, KS, JP, AE, LMU and LS completed the formal analysis. MH, KS and JP created initial draft of manuscript. MH, KS, JP, AE, LMU, and LS all edited the final manuscript.

All researchers identify as female, with 3 members representing mixed races/ethnicity (2 White/Latina, and 1 Asian/White), and 3 identifying as White. All have personal experience breastfeeding their children. These attributes of research team members were thoughtfully considered throughout the analysis process.

## Results

In total, 13 BFLM physicians from across the U.S. participated in the three focus groups, with each group including 2–6 participants. The focus groups included physicians from the following specialties: pediatrics (n = 10), family medicine (n = 2), and obstetrics and gynecology (n = 1). Illustrative quotes were drawn in relative proportions from the three focus groups with the greatest number participants and quotations from focus group 1 (FG1 – n = 5), followed by focus group 2 (FG2 – n = 6), and lastly focus group 3 (FG3 – n = 2).

All participants were female, with the majority being over the age of 40 (85%) and White (83%). Most were International Board Certified Lactation Consultants (IBCLCs) (92%) practicing in outpatient settings (42%), inpatient settings (17%), or both (42%), and primarily within academic healthcare systems (75%). Three quarters of the participants (75%) reported serving as directors of lactation, and 25% did not report holding an official lactation-related title within their institution. One participant did not respond to the demographic questions, leaving their information unknown.

### Importance of health systems in breastfeeding care

Participants emphasized the important role of health systems in implementing breastfeeding support practices and programs, underscoring their considerable power across the broader health ecosystem (see supporting quote “Q1” of Table 1 - T1-Q1). They stated that most physician education and training occur in health systems, so it is incumbent upon health systems to incorporate learning in BFLM for physicians (T1-Q2). As health systems are the major providers of health services, participants viewed health systems as uniquely positioned to significantly influence breastfeeding outcomes by implementing evidence-based practices (T1-Q3, T1-Q4). Finally, the historical role of health systems in perpetuating racial and social health disparities in the US was identified as a reason why these institutions must take responsibility for rectifying these injustices as related to breastfeeding (T1-Q5, T1-Q6).

**Table 1:** Example quotes related to the importance of health systems in breastfeeding care.

Q1: “Health systems are leaders and role models. And, they also have a fair amount of political influence and positions of power to implement changes in policy” (FG1) Q2: “Much of our physician training comes within the big health systems. If physicians aren’t trained on the importance and the foundation of breastfeeding…then it’s not going to be reinforced through various generations. And I think health systems are leaders and role models.” (FG1) Q3: “It’s important for health systems to support breastfeeding because I think it’s a foundation of primary care and preventative health” (FG1). Q4: “It starts…where most babies are born, in the hospital. And the first thing that they do is feed and bond with their mom” (FG 2). Q5: “[There are] enormous disparities in [breastfeeding] support…because wealthy people are able to navigate the system much better than people of limited means” (FG 2). Q6: “[Health systems] historically undermine lactation, women’s health, and self-advocacy...there’s a moral burden... for health systems to be at the forefront of that research and work” (FG 1).

### Current breastfeeding care practices in health system

Participants reported on instances of both well-functioning and suboptimal breastfeeding care in health systems. The overarching theme that emerged from the focus groups was the absence of a current systems-based approach to implementing and sustaining evidence-based breastfeeding interventions in health systems throughout the trajectory of breastfeeding. Participants described this gap in evidence-based care spanning all areas of the health systems that interact with the breastfeeding parent-infant dyad, including care for both healthy infants and those with special care needs, inpatient and outpatient, and all disciplines. The subthemes and supporting quotes within each construct of the EPIS framework are provided in Table 2 and are described below.

**Table 2:** Themes and example quotes for current health system practices in breastfeeding care.

Theme	(Q)uotation Number: Example Quote (Focus Group Number)
**EPIS Construct: Individual Characteristics**	
Individual staff experiences	**Q1:** “There’s an invisible wall there that [staff] are very afraid to cross, and I think that stems a lot from their own baggage [from personal breastfeeding experiences]…their own personal guilt is going to feed that fear, and then they’re less likely to prioritize [supporting breastfeeding]. ” (FG 3)
Individuals not following evidence-based breastfeeding care	**Q2**: “I just had a meeting not too long ago, where I showed randomized control trials and AAP policy…[a]nd what I got from the head of our nursery and the head of neonatology was, ‘Yeah, I just don’t think so.’” (FG 2)
**EPIS Construct: Leadership**	
Breastfeeding Medicine Department/Division	**Q3:** “My dream would include a true lactation or breastfeeding medicine department that is its own thing… everybody knows it’s there and knows that it’s available for consult. ” (FG 1) **Q4:** “We were lucky to form our own division [of BFLM]...which is going to afford us our own budget line, our own funding opportunities, fundraising opportunities, additional sort of structural support, administrative, secretarial, and so forth.” (FG 1) **Q5:“**A true lactation department that is multi-disciplinary, but it is really its own thing…with real muscle behind it…that the neonatology department looks on with respect. Like, ‘The breastfeeding medicine doctors think we need to manage our feeding protocol this way. Let’s listen to them.’” (FG1)
**EPIS Construct: Organizational Characteristics**	
Coordination of care	**Q6:** “There needs to be more synergism across [pediatrics and obstetrics] and inpatient and outpatient. …So I know that when they were discharged the baby had not had a good latch, and the mother still was having delayed lactogenesis, then I could help her differently in my newborn visit.” (FG 2)
Separation of infant and parent in care related to lactation	**Q7:** “The system’s not working. Ped[iatrics] and OB[stetrics] and Family Medicine… they have their separate silos, and the finances aren’t integrated. So, [they] have lactation support in the Peds office, but they can’t treat the mom.” (FG 1) **Q8:** “There’s no structure in our postpartum care for lactation. Everyone expects the baby to be seen 48 hours after discharge … and the mom at her [6 week] OB[stetrics] visit…lactation isn’t part of routine, after delivery care.” (FG 2)
Utilization of evidence based lactation practices	**Q9:** “There are still huge barriers, and it’s shocking how many [barriers] you can come across in a regular day. So, we’re not Baby Friendly.” (FG 1) **Q10:** “They’re constantly being told that just feed the baby till they can’t feed anymore…instead of being an evidence-based thing, it’s kind of this…which is really unbelievable.” (FG 2)
**EPIS Construct: Organizational Staffing Processes**	
Lactation staffing and pay equity	**Q11:** “[Lactation] is a service that is grossly understaffed for the size of the unit that we’re in. So, the 70 bed NICU [neonatal intensive care unit] has 3.0 FTE [full-time equivalents] lactation special specialists and my 18 bed unit has 0.0 FTE lactation specialists.” (FG 1) **Q12:** “LCs every day of the year, 12 hours a day…we’ve gone by the ILCA’s staffing recommendations for how to staff a large hospital, and they’ll go anywhere in the hospital.” (FG 1) **Q13:** “Pay equity for lactation consultants is an ongoing headache that results in understaffing and under service to our patient population.” (FG 1)
Insufficient clinical supervision	**Q14:** “Our lactation consultants are not one group. Not only do I have no supervision of them, but the NICU lactation consultants and the outpatient, and the education and the postpartum, and [labor and delivery], all work for different groups and there’s no standardization.” (FG 2) **Q15:** “The lactation consultants have no supervision…I have tried to get a capacity to oversee because I think it’s a risk management issue, it’s an ethical issue…But there is nothing in 15 years that I’ve been there...” (FG 1)
Workforce policies to support breastfeeding	**Q16:** “We found out there’s no policy at the Children’s Hospital for pumping and returning to work. So, we have employees that are having to do different things. Some clock out to pump, some don’t have extended hours to pump, some are required extended hours, and that’s all over the same health system.” (FG 2) **Q17: “**We do have a very large number of lactation rooms and we’ve added new ones. We have a breastfeeding policy for staff. We have a breastfeeding policy for trainees. We’ve gone through the ACGME to try to implement it and uphold that policy.” (FG 3)
**EPIS Construct: Training and Staff Education**	
Demand for trainee education on breastfeeding	**Q18:** “And so, our residents… they struggle with getting the breastfeeding knowledge that they want…when it comes to trying to improve the practice and the care that we’re providing our patients at the resident level, it inevitably is going to fall short, which is very frustrating just because of our lack of resources, that lack of education of our staff, so it becomes a perpetual cycle...” (FG 3)
Poor knowledge limits implementation of evidence-based practices	**Q19:** “All the OB[stetrics] residents rotate with me now. They have been really enthusiastic, because now I understand the terrifying total lack of any education [in breastfeeding] that OB[stetric]s have had in this country. …When I see OB[stetrics] notes there’s never a breast exam, in the hospital or the postpartum note, which once again I think it’s malpractice.” (FG 2)
**EPIS Construct: Quality & Fidelity Monitoring**	
Electronic medical records (EMR) impact implementation of breastfeeding support practices	**Q20:** “For the first 5 years of my career, I could not even tell you what our breastfeeding discharge rates were for our human milk discharge rates were in the NICU ever…And that recently changed…which means, I can tell what happens within my NICU unit itself. But anything that happens in labor and delivery, on the postpartum floor, or in newborn nursery, I still cannot access at all.” (FG 3)

#### Individual characteristics and leadership.

Healthcare professionals’ personal experience with breastfeeding, either positive or negative, was shared by participants as often affecting the breastfeeding care that is provided to patients (T2-Q1). Failure of providers and entire units to follow evidence-based medicine or implement evidence-based breastfeeding interventions was identified as a significant problem in all focus groups (T2-Q2). Some participants worked within divisions or centers for breastfeeding medicine which facilitated more widespread implementation of optimal breastfeeding support across their health systems. All participants agreed that the establishment of BFLM departments/divisions would provide the infrastructure to oversee the implementation of best practices in breastfeeding support, coordination of care, training, and clinical care for breastfeeding patients (T2-Q3, T2-Q4, T2-Q5). Participants highlighted the importance of integrating breastfeeding leadership within other departments, including those less directly connected to perinatal care but which may care for lactating women or breastfeeding dyads, such as surgery or the emergency room, and designating a “champion” in each unit or department.

#### Organizational characteristics, structures and staffing.

Participants noted that quality of breastfeeding care is impacted by coordination – or a lack thereof – between departments and levels of care (T2-Q6). For instance, in some health systems improved access to hospital delivery records enhanced the continuity of care and facilitated breastfeeding support at the outpatient newborn or postpartum visit. Participants recognized that the siloing of lactation care within different departments further complicated efforts to support the breastfeeding dyad (T2-Q7, T2-Q8). Participants discussed shifting towards a model that included joint comprehensive lactation care for the parent-infant dyad upon discharge. While standards exist, participants highlighted that evidence-based policies were not implemented due to the overwhelming numbers of barriers within the health systems, which impacted lactation care. (T2-Q9, T2-Q10).

Insufficient number of lactation staff for clinical need and patient volume was expressed by participants (T2-Q11), while others had robust lactation staffing following national guidelines (T2-Q12). Staffing of lactation was further complicated by poor pay (T2-Q13). Lactation staff were found to be dispersed across different departments, undermining cohesion and coordination (T2-Q14). Participants cited that lack of clinical supervision for lactation staff creates risk and impacts the quality of lactation support (T2-Q14, T2-Q15).

#### Staff education and quality and fidelity monitoring.

Participants described highly variable policies and benefits for lactating employees. Some health systems had insufficient resources and support for employees, while others had significant infrastructure and had implemented national standards for support of lactating medical trainees (T2-Q16, T2-Q17). Participants emphasized a lack of adequate breastfeeding education for residents, highlighting gaps in knowledge among residency faculty and insufficient resources, which perpetuates a cycle of inadequate care (T2-Q18). A notable example was the lack of breast exams documented in obstetric notes, which was criticized as a serious oversight, bordering on malpractice (T2-Q19). The EMR was highlighted by participants as having significant limitations, such as the inability to access comprehensive breastfeeding data across departments, which hampers efforts to track key metrics (T2-Q20).

### Recommendations for optimal breastfeeding care in health systems

**Fig 1:**
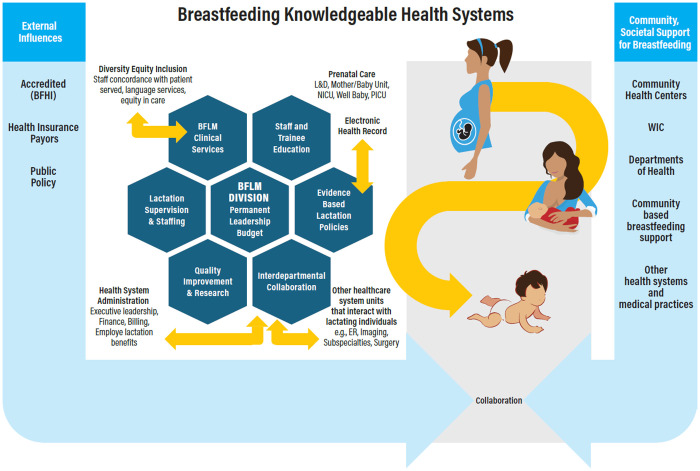
Recommendations for Breastfeeding Knowledgeable Health Systems (BKHS). This figure illustrates the multilevel structures required to deliver high‑quality, equitable breastfeeding care, aligned with the eight recommendations in Table 3, and describes outer context and inner context factors. Arrows represent bridging factors that connect these domains as the system supports the dyad from pregnancy through postpartum and beyond. BFLM Breastfeeding and Lactation Medicine; BFHI Baby-Friendly Hospital Initiative; L&D Labor and Delivery; NICU Neonatal Intensive Care Unit; PICU Pediatric Intensive Care Unit; ER Emergency Room; WIC Special Supplemental Nutrition Program for Women, Infants, and Children.

Focus groups resulted in 8 key recommendations for BKHS, summarized in Table 3 and illustrated in Fig 1, reflecting many of the optimal practices described above. Member checking from survey respondents (12 out of 13) to confirm the recommendations resulted in near unanimous agreement.

**Table 3:** Recommendations for optimal breastfeeding care in health systems.

A health system that is providing optimal breastfeeding care and management should: 1. Integrate evidence-based, full-spectrum breastfeeding care and management for breastfeeding families across the entire health system. 2. Provide breastfeeding support that is accessible and equitable for all patients, particularly for historically minoritized or vulnerable populations. 3. Create and maintain permanent, funded leadership positions and structures that are responsible for clinical and administrative oversight of breastfeeding services across the health system, such as a division or department of breastfeeding medicine. 4. Educate all staff and trainees on all breastfeeding care and management as relevant to their discipline and duties. 5. Implement policies, procedures and services that comprehensively support breastfeeding employees. 6. Implement systems of monitoring and quality improvement to support all breastfeeding-related programs and clinical outcomes. 7. Incorporate breastfeeding status and related care into the electronic health record. 8. Collaborate with community breastfeeding support programs to ensure adequate support and resources for the patient population served.

### Barriers and facilitators to implementing optimized breastfeeding care

Participants reported barriers and facilitators that were both internal and external to the health system, representing themes in both EPIS internal and external contexts (Table 4).

**Table 4:** Themes and example quotes for barriers and facilitators to implementing optimized breastfeeding care.

Theme	Quotation Number: (Barrier/Facilitator) – Example Quote (Focus Group Number)
**EPIS INNER CONTEXT**	
**EPIS Construct: Individual Characteristics**	
Individual attitudes, self-efficacy and knowledge regarding breastfeeding can serve as barriers or facilitators to health system change	**Q1: (Barrier) -** “I still think there’s a large cohort of providers who did not have a successful breastfeeding experience, and that does taint their approach to things. Unfortunately, this becomes a really personal thing. I had a newborn nursery director, said “MB, I’m not going to die on a sword for breastfeeding, because it didn’t work out for me.” (FG 2) **Q2: (Facilitator) -** “Almost every single advance I’ve made and my breastfeeding department is because I met somebody with a personal [breastfeeding] experience where they’re like, ‘If I had known you.’ I mean, literally last month the guy from purchasing.” (FG 2)
**EPIS Construct: Leadership**	
Leadership – both the people in power and the roles and leadership structure – influence the environment and policy regarding patient breastfeeding services and health system change	**Q3: (Barrier)** “I know that the CEO and the Media Vice President are two women who did not breastfeed their children, and so I hate to say this, but the minute you say breastfeeding sometimes, you can tell other people did it themselves or not...” (FG 2) **Q4: (Facilitator)** “And so people that have been barriers to me that I didn’t know, they were invisible until finally I tracked down the right person were compliance, managed care contracting, legal, revenue cycle...we need all of those people...” (FG 3)
The profession of medicine has not incorporated breastfeeding and lactation as part of its domains	**Q5: (Barrier)** “...there’s this gulf between nurses and doctors at a lot of health systems. And in a lot of health systems that were not physician led, traditionally…” (FG 2) **Q6: (Barrier)** “In medicine we kind of gave human lactation away. It’s almost like we’re not that interested here. Let’s just let somebody else take It over.” (FG 3)
**EPIS Construct: Organizational Characteristics**	
The culture of the institution impacts the prioritization of breastfeeding and implementation of new practices	**Q7: (Barrier)** “I feel like there’s a culture that I battle every day of ‘it’s somebody else’s job to deal with mom’s milk.’ (FG 1) **Q8: (Facilitator)** “I was excited to come here because I don’t have to talk about “why” anymore; I just talk about “how” all day long.” (FG 1)
Siloing of services, infrastructure and staff in different departments impacts the ability to implement care for the breastfeeding dyad	**Q9: (Barrier)** “...because lactation grew up organically and they’re sort of a champion at different places…It could be a wonderful multidisciplinary specialty but instead, we’re really fragmented…” (FG 2) **Q10: (Facilitator)** “And I have a great partner in ENT. We have a lot of traction with our Occupational Therapy department. And right now we’re very fortunate that either through nutrition…that sort of group of clinically relevant, related people is also really good because that’s those are the relationships we’ve been building over the years.” (FG 2)
**EPIS Construct: Organizational Staffing Processes**	
Institutional policies impede hiring of lactation professionals with racial/ethnic concordance with patients	**Q11: (Barrier)** “We have this local IBCLC; she’s a woman of color…. And it takes 9 months to figure out how to get her a badge.” (FG 1) **Q12: (Barrier)** “Recently I was able to hire the only Black lactation consultant in [our state]…[I]t took a lot because our hospital only will have nurse lactation consultants, and so we hired her as a health educator.” (FG2).
Support for lactating employees impacts their breastfeeding experience, which ultimately influences employee attitudes toward providing breastfeeding care	**Q13: (Barrier)** “If one person is working on one hospital floor, like a staff nurse, she might have to go to the other end of the hospital in order to access the lactation room… it’s difficult for health systems to support patients and parents if the staff is frustrated with their own lactation experience.” (FG 3)
**EPIS Construct: Quality & Fidelity Monitoring**	
Quality improvement and clinical data monitoring impact implementation of evidence based breastfeeding practices	**Q14**: **(Facilitator)** “So [health system name] provides care across the lifespan and pediatrics is a small part that doesn’t make them a lot of money. But we do have armies of people that look at Medicare benchmarks. And so the QI people have been helpful too…it would be great to have more benchmarks because we’ve got a lot of people within the system that are interested in these.” (FG-2) **Q15: (Facilitator)** “I think that within our area one reason Baby Friendly, happened is because it was a measurable concrete thing people could go through and then they could advertise they had achieved it. If you can kind of create some of these incentives and be able to track those measures so people can get that feedback, it’s really helpful.” (FG 1) **Q16: (Facilitator)** “...because the health systems tend to involve massive electronic medical records… that is a place we can build out the prompts, hard stops, forced pathways that actually make change. And, that sounds so mechanical and cold, but, in my experience…[y]ou can’t educate or convince people…changing the electronic medical record has proven to be the most effective thing in my career, thus far” (FG 1)
**EPIS Construct: Infrastructure, Financial Support & Resources**	
Funding, resources and financial implications for health system of breastfeeding care is variable and misunderstood	**Q17: (Barrier)** “Right now, I’m running these services, and I get inadequate reimbursement at the physician level and inadequate reimbursement at the LC [lactation consultant] level. And I’m told to create business plans that are budget neutral, which is impossible when you’re paying an RN IBCLC [International Board Certified Lactation Consultant] 30–40 bucks an hour, and you get reimbursed at 18.” (FG 1) **Q18: (Facilitator)** “I think also there’s a misunderstanding that this type of prevention comes at cost to health systems. Right like, that you can’t make money doing human lactation support or breastfeeding support, and I think that’s not correct.” (FG 3)
**EPIS OUTER CONTEXT**	
**EPIS Construct: Funding**	
Payors and insurance companies can facilitate or hinder implementation of BFLM care	**Q19: (Barrier)** “The fact that the Affordable Care Act hasn’t been implemented very well is particularly irritating to me on the front of prenatal education, because it’s supposed to be a preventative care benefit. And the fact that people cannot get insurance to pay for prenatal classes is in large part, in my area, because our hospital system hasn’t made the effort to figure out how to bill insurance.” (FG 1) **Q20: (Facilitator)** “[Our breastfeeding clinic] make[s] a lot of money. So I think that helps because we bill insurance for the mom and the babies...you know money talks as I’m sure you guys have talked about. So we have private insurance that pays for our breastfeeding clinic.” (FG 1)
**EPIS Construct: Patient/Client Characteristics**	
Patient characteristics, language, and social determinants of health make implementing breastfeeding care more challenging.	**Q21: (Barrier)** “Making sure that our patients are getting served in the language that they best understand, especially that you know night or 2 out, that first night or 2 out after having a baby, you’re exhausted you know, you’re crying, the baby’s crying. It just really helps to have somebody who actually speaks your language, or is willing to get an interpreter which it doesn’t always happen, unfortunately.” (FG3) **Q22: (Facilitator)** “I think we have a very ethnically and culturally diverse population; although people may not realize that, I would use routinely 15 to 20 different interpreters every morning on my newborn nursery routes… We worked early on and trying to train doulas from many different backgrounds. It’s just really hard to have the time to do everything” (FG1)
Societal values and norms around breastfeeding and maternal child health impact implementation	**Q23: (Barrier)** “lactation just has a lower priority, and I don’t know if that is because, as a society, we have still have it wrapped in this whole breastfeeding is natural thing, therefore we don’t need to think about it or focus on it, or research it as much as anything like everything else.” (FG 3) **Q24: (Facilitator)** “I think our culture in general is changing again in a way to support human lactation and human milk. And I think medicine is changing. I think my residents are really fascinated by it. They really are just so excited to be in lactation clinic with me, and then round on OB[stetrics] with me to do that extra check like, let’s latch every baby on postpartum while we go through.” (FG 3)

#### Individual characteristics and leadership.

The personal breastfeeding experiences of staff were noted by participants as potential barriers (if negative) or facilitators (if positive, or if interpreted as a desire to help others) to the adoption of breastfeeding-supportive policies (T4-Q1, T4-Q2). They expressed that finding the “right” people in leadership with positive attitudes toward or personal experiences of breastfeeding was required for crafting and implementing health system practices (T4-Q3, T4-Q4). At the clinical level, participants shared that breastfeeding problems were not always seen as medical concerns, hindering efforts to incorporate BFLM into health systems. (T4-Q5, T4-Q6).

#### Organizational characteristics, structures and staffing.

Participants described centralized breastfeeding medicine leadership as a facilitator of health system transformation. In organizations where policies were not overseen by a BFLM department or division, there was a lack of prioritization of breastfeeding (T4-Q7) and siloing of care (T4-Q9), but in health systems where BFLM was championed, the culture of the organization was transformed (T4-Q8) and multidisciplinary groups of specialties and departments worked together synergistically to support breastfeeding and lactation (T4-Q10).

Participants also shared that hiring policies, such as requiring lactation consultants to also have nursing degrees, limited the ability of organizations to hire lactation professionals, particularly those that have racial/ethnic or cultural concordance with patients. (T2-Q11; T2-Q12). In systems without supportive employee lactation policies, participants reported that staff’s personal breastfeeding experiences were likely to be poor, exacerbating barriers to health systems change (T4-Q13).

#### Quality and fidelity monitoring, infrastructure, and financial resources.

Measurable benchmarks and incentives were reported to drive health systems to make improvements in breastfeeding support. For instance, Baby-Friendly certification succeeded in one institution for this reason (T4-Q14, T4-Q15). The capacity of the EMR to drive systemic change in breastfeeding support was noted by participants. Built-in prompts, hard stops, and forced pathways within EMRs were identified as effective tools to standardize practices and ensure compliance, often proving more impactful than education or persuasion alone (T4-Q16).

Participants noted that underbilling for lactation care created fiscal barriers to implementing adequate breastfeeding support (T4-Q17), and that when billing appropriately for both members of the dyad, breastfeeding and lactation support could be profitable for institutions (T4-Q18).

#### Funding and patient characteristics.

Participants reported that inadequate implementation of the Affordable Care Act hindered access to prenatal education, due to insufficient billing for these services (T4-Q19), but some shared that clinics thrived by billing private insurance for both parent and infant care, demonstrating sustainability potential (T4-Q20). Providing culturally appropriate lactation care in multiple languages and directed recruitment efforts for concordant staff (T4-Q22) were identified as critical by participants, but often underprioritized (T4-Q21). Participants reported that societal norms regarding the perceived “ease” of breastfeeding often undervalued lactation needs (T4-Q23), though they cited cultural shifts recognizing its importance (T4-Q24).

## Discussion

This is the first study to explore physician perspectives on the delivery of optimal breastfeeding care and support within large health systems. BFLM physicians in the focus groups highlighted their perspectives on the leadership role which health systems should play in the delivery of breastfeeding services within the US and the training of the healthcare workforce. From the study, we identified strategic implementation recommendations from BFLM experts for optimal breastfeeding support in health systems and barriers and facilitators related to the implementation of BKHS. These recommendations (Table 3) and the model (Fig 1) of health system breastfeeding services and structures provide a practical framework for those who endeavor to develop and implement a BKHS [41], outlining key domains and contextualizing the health system within the socioecological system of breastfeeding [42].

To deliver integrated, evidence-based, full-spectrum breastfeeding and lactation care within health systems, participants in our study recommended permanently funded medical director positions within BFLM departments, including protected time for education and research, in line with other studies [30,32,43,44]. Currently, few BFLM divisions or centers exist in the U.S. with only one division of BFLM formally incorporated within a medical school in the U.S. [32,45,46]. Coordination of breastfeeding medical care can be strengthened through system-wide breastfeeding-support policies developed collaboratively by key stakeholders, including clinical departments, health system leadership and administration, and community partners [12].

Respondents in our study suggested that health system capacity can be addressed by adopting existing US Lactation Consultant Association staffing guidelines for IBCLCs in inpatient postpartum hospital units, neonatal intensive care units, and post-discharge care [47]. New research is needed to update recommendations for appropriate staffing ratios of the growing interdisciplinary breastfeeding workforce, including BFLM physicians, other BFLM providers, lactation consultants and trained peer and community support.

Similar to previous reports, our respondents described that clinicians who have had negative experiences with breastfeeding may be less likely to prioritize lactation support and may apply their personal experiences in place of evidence-based medicine [48,49]. Our study extends this to health systems leaders, whose personal breastfeeding experiences were noted to influence system-wide policy, decision-making and resources for breastfeeding support. Breastfeeding mothers want evidence-based care from their physicians and other providers [50], however the personal breastfeeding experiences of healthcare providers has been shown to play a major role in breastfeeding support [51]. Increased support for lactating employees and evidence-based lactation training for health systems’ staff and clinicians has been shown to improve patient breastfeeding outcomes [52–56]. Lactation education also improves staff attitudes, confidence, and competence [52,57,58].

BFLM physician leaders in our study recommended that quality improvement and tracking of breastfeeding status within the EMR may provide opportunities to implement breastfeeding support practices within health systems. Although there can be inconsistencies in the way that lactation data are recorded in the EMR [59] and challenges in connecting parental and infant records [60], EMRs might affect change by tracking breastfeeding metrics [61], monitoring key indicators like rates of human milk feeding and skin-to-skin contact, creating standardized workflows to promote evidence-based breastfeeding practices, and instituting algorithms and security mechanisms to alert clinicians and staff to situations of risk [62]. Improved documentation through the use of the EMR may enhance lactation care reimbursement by insurance providers and health systems payors [63]. The need for tracking breastfeeding data including the identification of breastfeeding issues prior to discharge extends beyond healthcare institutions to the community. The National Association for County and City Health Officials included the need for a shared database system in the Community Infrastructure recommendations as a means to advance chest/breastfeeding continuity of care [64].

The negative effect of racism and implicit and explicit bias on breastfeeding outcomes has been described in the literature and was highlighted by our respondents [65,66]. The lack of diversity among lactation consultants and breastfeeding medicine physicians and other providers, the vast majority of whom are non-Hispanic White and are fluent only in English [63], precludes staff-patient racial or ethnic concordance in many health systems. Ensuring breastfeeding support that is truly accessible and equitable for all patients requires diversity, equity, and inclusion training for all lactation and BFLM staff, along with health system hiring practices that promote racial, ethnic, and cultural concordance between clinicians and patients [61]. This includes expanding access to IBCLC training opportunities and eliminating additional nursing certification requirements when hiring lactation consultants. Collaboration with community breastfeeding support programs plays an important role in achieving continuity of care and increasing breastfeeding exclusivity and duration in the surrounding communities of health systems, especially in minoritized groups with historically low breastfeeding rates [41,67–70].

Our findings highlight the complexity of integrating a new clinical care model into health systems. The integration of BFLM has similar opportunities and challenges to the field of addiction medicine, as both require care that extends into a variety of clinical settings from inpatient to outpatient, surgery to ICU and beyond. Popular breastfeeding misconceptions, such as breastfeeding is “natural and easy,” are believed to undermine medical concerns and disease processes. A paradigm shift to legitimize and prioritize BFLM within health systems is felt to be long overdue [71]. The focus group participants emphasized that it is time for the development of new patient care models, increased research into the pathophysiology of breastfeeding and lactation, and an understanding of how to best deliver BFLM care within health systems to the breastfeeding dyad. Health systems are also best positioned financially to negotiate appropriate compensation with insurers for services, including those for breastfeeding and lactation, across the entire system [72,73]. With large health systems providing the majority of medical care within the US [74], the implementation of evidence-based practices, policies and leadership structures within these entities is believed to be essential to ensuring optimal breastfeeding and lactation outcomes.

Next steps for this research include surveying BFLM physicians to identify BFLM divisions and health systems already implementing some of the best practices described here. Further research must also engage other health system stakeholders, including administration, finance, and nursing leadership, to better recognize administrative and financial factors in the development of BKHS.

### Strengths and limitations

Strengths of this study lie in our ability to explore the knowledge and experiences of highly accomplished physician leaders in this field and the use of the EPIS framework to understand and guide the implementation of a BKHS. Limitations of this study relate to the small sample size and homogeneity of participants, where collected perspectives may not represent all possible viewpoints within BFLM, including representativeness of the sample potential biases, and limits of saturation. This study included a sample of just 13 participants, all of whom held leadership positions in breastfeeding and lactation medicine. At the time of manuscript writing, only 159 individuals were publicly listed as board-certified by the North American Board of Breastfeeding and Lactation Medicine, which began board certification in 2023 [75]. A recent US-wide survey identified 138 physicians and other providers practicing breastfeeding and lactation medicine [76]. Given the small and specialized nature of this professional community, the high proportion of representation enhances the relevance and depth of the study findings.

The homogeneity of the sample, recruited from among subjects who already had a professional relationship with study team members, may not fully encompass the diversity of BFLM physicians across demographic backgrounds, geographic regions, and health system types. This may have skewed the discussion in our focus groups and introduced potential bias, thereby limiting the generalizability of findings to more varied clinical contexts, especially among non-white, non-physician led BFLM departments/divisions and at non-academic, community-based health systems. However, even among this small and specialized group, most focus group participants did not have prior relationships with one another. Saturation was not assessed due to the limited population size, and the study aimed to capture a sample of expert perspectives rather than achieve thematic saturation. Future work will expand the breadth of BFLM providers and health systems surveyed.

## Conclusion

Many barriers exist for the delivery of optimal breastfeeding care within health systems. In this study, we identified eight recommendations to facilitate systemic changes in large health systems as well as important implementation barriers to overcome, such as leadership and staff personal breastfeeding experiences impeding evidence-based care. These elements are intended to guide strategic planning and prioritization by offering a structured yet adaptable roadmap that health systems may tailor to their organizational context and readiness. The creation of a BKHS requires integrated breastfeeding care and management across entire health systems maintained and led by a dedicated BFLM division headed by an experienced clinician to ensure the implementation, monitoring, and quality improvement of breastfeeding supportive policies for all patients and employees in collaboration with community programs.

## Supporting information

S1 Table Definitions of domains and constructs within exploration, preparation, implementation, and sustainment (EPIS) framework. (DOCX)

S2 Table Focus group facilitator guide. (DOCX)

S3 Table Selected codes within exploration, preparation, implementation, and sustainment (EPIS) framework. (DOCX)
