# Supplementary material for: Building breastfeeding knowledgeable health systems: Focus groups with physician leaders
Source: PLoS One. 2026 May 28;21(5):e0350146. doi: 10.1371/journal.pone.0350146 (PMC13218481; doi:10.1371/journal.pone.0350146)
Supplement: S1 Table — (DOCX) [file pone.0350146.s001.docx]

| **S1 Table: Definitions of Domains and Constructs within Exploration, Preparation, Implementation, and Sustainment (EPIS) Framework.** | | |
| --- | --- | --- |
| **Inner Setting Domain:** The characteristics within an organization such as leadership, organizational structures and resources, internal policies, staffing, practices, and characteristics of adopters (clinicians or partitioners) | | |
|  | **Construct** | **Definition** |
| **Individual Characteristics** | | Shared or unique characteristics of individuals (e.g., provider, supervisor, director) that influence the process of implementation. |
| **Leadership** | | Characteristics and behaviors of individuals involved in oversight and/or decision-making related to EBP implementation within an organization. |
| **Organizational Characteristics** | | Structures or processes that take place and/or exist in organizations that may influence the process of implementation. (e.g. culture, structure) |
| **Infrastructure, Financial Support & Resources** | | Fiscal support provided by the system in which implementation occurs. Fiscal support can target multiple levels (e.g., staff training, fidelity monitoring, provision of the innovation/EBP) involved in implementation and delivery/use of the innovation. |
| **Organizational Staffing Processes** | | The processes or procedures in place at an organization relate to the hiring, review, and retention of staff involved in the active delivery of the innovation/EBP and/or its implementation (e.g. professional training, qualification related to EBI delivery) |
| **Quality & Fidelity Monitoring** | | Processes or procedures undertaken to ensure adherence to active delivery of the innovation/EBP and/or an implementation strategy. (e.g. Quality assurance evaluation) |
| **Outer Context Domain:** The environment external to the organization and characteristics of the individuals who are the targets of the evidence-based practice (e.g. patients) | | |
| **Funding** | | Fiscal support provided by the system in which implementation occurs (e.g. Fee-for-Service, grants) |
| **Patient characteristics** | | Demographics and individual characteristics of the target population/ end user. |
| **Bridging Factors Domain:** Factors that span the outer and inner contexts such as community-academic partnerships, and existing and developing relations between policy and health care provider organizations. | | |
|  | |  |
