# Supplementary material for: Building breastfeeding knowledgeable health systems: Focus groups with physician leaders
Source: PLoS One. 2026 May 28;21(5):e0350146. doi: 10.1371/journal.pone.0350146 (PMC13218481; doi:10.1371/journal.pone.0350146)
Supplement: S2 Table — (DOCX) [file pone.0350146.s002.docx]

**S2 Table. Focus group facilitator guide.**

| The role of the health system in breastfeeding support   1. Why is it important for health systems to support breastfeeding?   (Probe: Do they play a central role in supporting breastfeeding? If not, should they?)   1. How can health systems promote equity in breastfeeding?   Current Practices   1. What are examples of ways that health systems are currently facilitating breastfeeding? 2. What are examples of ways that health systems are currently preventing or causing barriers to breastfeeding?   Recommendations   1. If a health system were to be a center of excellence of infant feeding, what would the criteria be? (Probe: What qualities define a health system “lactopia”?) 2. Who would be the stakeholders who should sit at the table for making these types of changes at the health system level?   Barriers and Facilitators to Implementation   1. What are potential/expected barriers to change for health systems to support breastfeeding? (Probes: Readiness for change, knowledge/skills, financial, climate, leadership, i.e. who are the stakeholders?) 2. What facilitators are needed for health systems to become centers of excellence in lactation and breastfeeding? |
| --- |
