# Supplementary material for: Building breastfeeding knowledgeable health systems: Focus groups with physician leaders
Source: PLoS One. 2026 May 28;21(5):e0350146. doi: 10.1371/journal.pone.0350146 (PMC13218481; doi:10.1371/journal.pone.0350146)
Supplement: S3 Table — (DOCX) [file pone.0350146.s003.docx]

| **S3 Table: Selected Codes within Exploration, Preparation, Implementation, and Sustainment (EPIS) Framework.** | | |
| --- | --- | --- |
| **Inner Setting Domain:** The characteristics within an organization such as leadership, organizational structures and resources, internal policies, staffing, practices, and characteristics of adopters (clinicians or partitioners) | | |
|  | **Construct** | **Selected Codes** |
| **Inner Context Domain** | | |
| **Individual Characteristics** | | - Attitudes, knowledge and self-efficacy of staff in breastfeeding support - Staff personal breastfeeding experiences impact patient care |
| **Leadership** | | - Leaders’ attitudes and personal experiences of breastfeeding - Individual “breastfeeding champions” - Leadership in BFLM at institutional level |
| **Organizational Characteristics** | | - Culture of institution and people within it - Evidence-based practice not implemented in breastfeeding support - Importance of healthcare systems in breastfeeding |
| **Infrastructure, Financial Support & Resources** | | - Budget for breastfeeding services - Patient care billing and health system finances |
| **Organizational Staffing Processes** | | - Employee lactation support - Lactation staffing levels - Need for supervision of lactation clinical care/staff |
| **Quality & Fidelity Monitoring** | | - Electronic medical records as a tool in implementing evidence based - Need for data and monitoring of breastfeeding rates and care - Quality improvement initiatives |
| **Outer Context Domain** | | |
| **Funding** | | - Payors and insurance |
| **Patient characteristics** | | - Care of diverse populations |
| **Sociopolitical context** | | - Societal beliefs about breastfeeding |
| **Bridging Factors Domain** | | |
| **Interorganizational Environment** | | - Accreditations (e.g., BFHI) - Community organizations (e.g., WIC) |
| **Innovation Factors** | | |
| **Recommendations for healthcare systems for optimal breastfeeding support** | | - Provide evidence-based breastfeeding care - Sufficient staffing of lactation - Coordination of care of breastfeeding dyads across the healthcare system |
